# Knee megaprosthesis: a salvage solution for severe open and complex distal femoral fracture associated with an ipsilateral brachial plexus injury (a case report with literature review)

**DOI:** 10.11604/pamj.2015.21.207.7260

**Published:** 2015-07-20

**Authors:** Badr Ennaciri, Christian Vasile, Thierry Lebredonchel, Mohamed Saleh Berrada, Eric Montbarbon, Emmanuel Beaudouin

**Affiliations:** 1 Department of Orthopedics, Avicenna University Hospital, Rabat, Morocco; 2 Department of Orthopedics, Chambéry Hospital, Chambéry, France

**Keywords:** Knee megaprosthesis, femoral spacer, open fracture

## Abstract

Complex distal femoral fractures in the young patient often occur as a result of high velocity trauma. Timely recognition and treatment is everything in such a situation, and it needs a robust staged management pathway to optimize the chance of limb preservation. We report a case of a motorcyclist admitted to the department of orthopedics at Chambery hospital, France, with a complex comminuted and open distal femoral fracture of the left leg, associated with a brachial plexus injury to the ipsilateral upper limb. On arrival to the emergency department, damage control stabilization and surgery was commenced, debridement of contaminated non-viable tissue, abundant antiseptic lavage and application of external fixation coupled with the use of antibiotic spacer. Following normalization of inflammatory markers and ensuring no clinical signs of infection, subsequent management consisted of joint reconstruction to achieve a functional knee. The external fixator and femoral spacer was removed and a modular megaprosthesis was implanted with a lateral gastrocnemius flap to cover the exposed knee joint and reinforce the extensor apparatus. Nerve graft to the left brachial plexus injury was performed at University Hospital of Grenoble. Our patient entered an intensive rehabilitation program and at 1 year follow-up achieved good knee function and sensation to the left upper limb.

## Introduction

Distal femoral fractures are not uncommon and are often the result of high velocity road traffic collisions. The management of such injuries is challenging. Supra-condylar femoral fractures with bone loss and neuro-vascular involvement in the poly-traumatized patient demand emergency timely decisions, the aim being to preserve the limb, avoiding amputation. Considerations in the management of these injuries are addressing the open fracture, method of skeletal stabilization, soft tissue coverage and timing of soft tissue coverage and strategies for bone grafting [1]. The aim of this case report is to describe our damage control guideline in the management of open femoral fractures with huge bone and soft tissue loss, associated with an ipsilateral upper limb palsy. Secondarily we will describe the further operative procedures performed to restore knee function.

## Patient and observation

**Figure 1:**
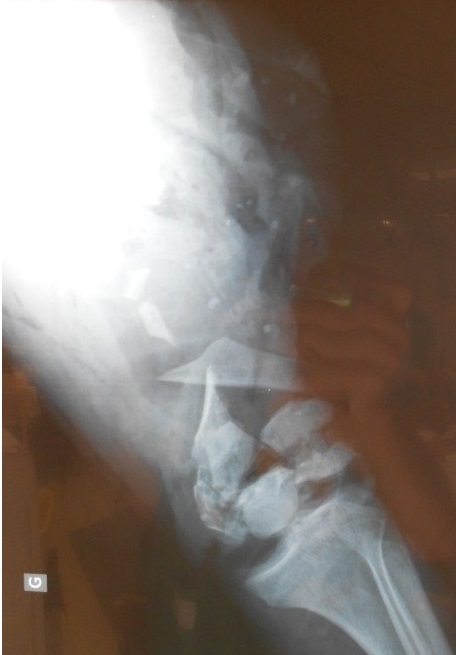
Anteroposterior radiograph of the left knee showing comminuted distal femur fracture with bone loss

**Figure 2:**
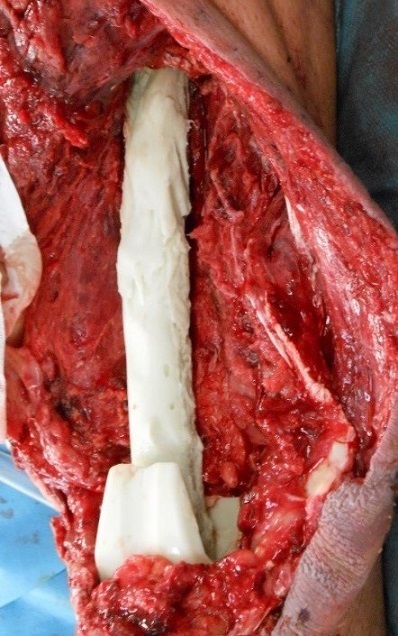
Surgical view of the femoral spacer

**Figure 3:**
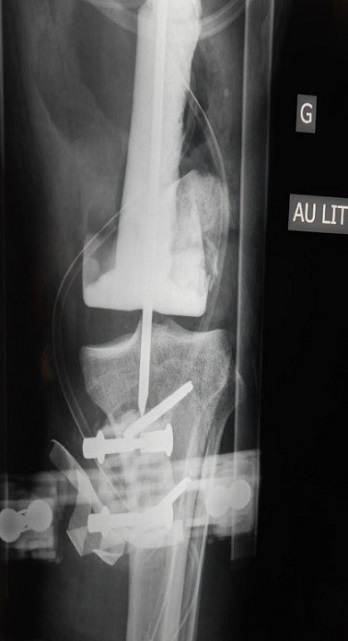
Anteroposterior radiograph of the left knee showing femoral spacer with external fixator

**Figure 4:**
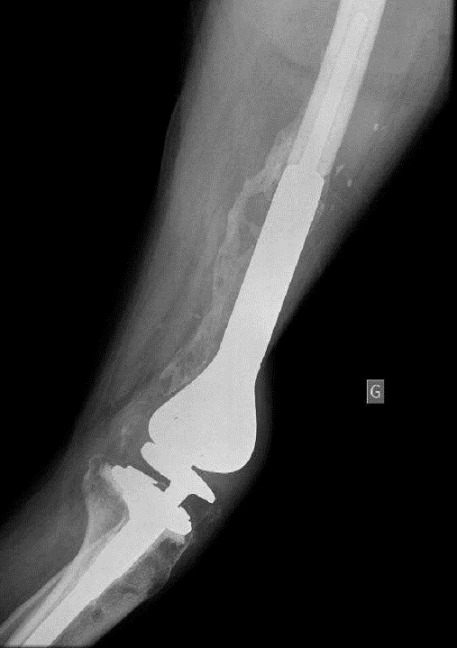
Lateral radiograph of the left knee showing pseudo-synovial membrane ossification around the knee megaprosthesis

**Figure 5:**
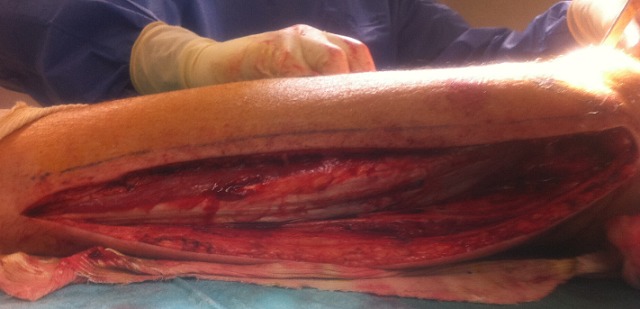
Lateral approach of the left leg exposing the lateral gastrocnemius muscle

**Figure 6:**
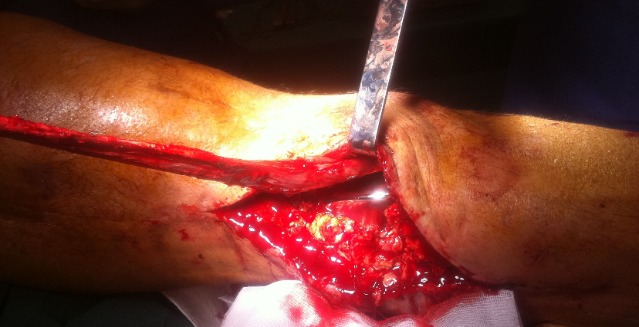
Lateral gastrocnemius flap was passed to cover the exposed knee

**Figure 7:**
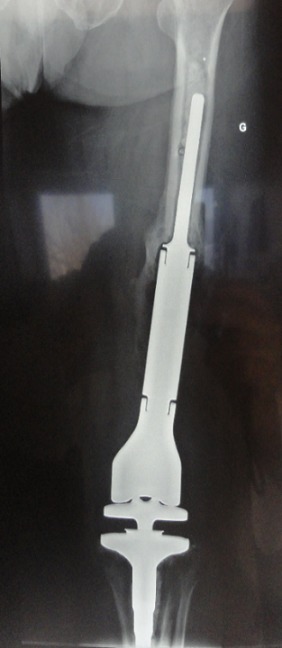
Anteroposterior radiograph showing good osseointegration of the megaprosthesis one year after knee replacement

A 46 year old man was admitted to the emergency department at Chambery hospital, France, one hour after a high velocity traffic accident (mechanism of injury, the patient a motorcyclist collided into a vehicle). He was in hemorrhagic shock on arrival. He had sustained a comminuted open distal femoral fracture (Gustilo-Anderson classification 3C, (Figure 1). Neuro-vascular status was normal to the left leg. Objective neurological examination revealed a left upper limb palsy. After a period of stabilization in the intensive care unit, the patient was transferred to the operating room, where hemostasis was addressed with electrocautery, concurrently with extensive debridement of non-viable tissue and abundant lavage. The limb was stabilized with a bridging knee external fixator (Hoffman II) femoral-tibial application. Topical Negative Pressure dressing was applied to the distal thigh. 48 hours later, further debridement of non-viable tissue was performed and a femoral spacer placed to the dead space. It was necessary to remove the femoral spacer 10 days later due to local sepsis as a result of enterobacter cloacae and pseudomonas aeruginosa. This was treated with intravenous antibiotic therapy. Another femoral spacer was placed after 45 days (Figure 2 and Figure 3). 4 months following the injury, and once infection was controlled, at a single operative stage the external fixator and femoral spacer were removed, and a knee megaprosthesis GMRS cemented (Global Modular Replacement System) was implanted with additional reinforcement of the patella tendon using synthetic ligament. Full ambulation was achieved before the patient was discharged from hospital (Figure 4). 45 days after the implantation of the megaprosthesis, the patient presented with extensive necrosis of the extensor apparatus. This necessitated debridement and patellectomy. 10 days following the patellectomy, a lateral gastrocnemius flap was used to cover the exposed megaprosthesis and restore the extensor mechanism (Figure 5 and Figure 6). The left leg was immobilized in plaster for 45 days to protect the flap. Nerve graft to the left brachial plexus injury was performed at the University Hospital of Grenoble. The patient received daily physiotherapy to develop the strength and range of motion of the upper limb (passive mobilization and electrostimulation). Good results were obtained at one year after the definitive soft tissue coverage with the lateral gastrocnemius flap. International Knee Society Score was 79. Sensation was restored to the upper limb. The patient was able to walk, squat and accomplish daily activities without great difficulty. There was 1.5 cm of shortening to the left leg. The anteroposterior and lateral radiographs revealed good osseointegration of the megaprosthesis (Figure 7).

## Discussion

Serious limb injuries can occur as a result of high velocity trauma, as a result of road traffic accidents or in the workplace. Comminution and open injuries can indicate high velocity trauma. This can also be associated with other bony injury and fatal visceral injury [2]. In the management of our patient, after lavage, extensive debridement of non-viable tissue, abundant antiseptic lavage; external fixation coupled with antibiotic femoral spacer was very important to stabilize the patient and the knee. Major vascular and nervous injury in the knee was not present. Donegan et al. demonstrated successful use of antibiotic spacers for patients presenting with acute open fractures with bone loss and those patients who had chronic infection and non-union to the femur and tibia; antibiotic spacers may sterilize a wound bed and provide a useful scaffold for incorporation of bone graft [3]. Ostermann and al. also describe the usefulness of local antibiotic therapy in a series of Gustilo-Anderson grade I-IIIb upper and lower extremity fractures with a decrease in chronic infection rates from 12% to 3% [4]. The implantation of a megaprosthesis should be performed after normalization of inflammatory markers, and when there is no sign of infection [5]. The removal of the spacer should reveal evidence of a pseudo-synovial membrane that provides the prosthesis with a sterile and protected environment [6]. In this case, induced membrane ossifications (Figure 4 and Figure 7) have made the megaprosthesis more stable.

The choice of megaprosthesis was an interesting alternative with the aim to preserve some quality of life for our patient. The modularity of such a device enables good restoration of the limb length at the same time avoiding iatrogenic injuries related to the stretching of major neurovascular structures [6]. A complex soft tissue defect resulted from trauma, infection and complications of total knee arthroplasty; the extensor mechanism of the knee is vulnerable in such situations. Patella tendon deficiency and the exposed joint must be addressed simultaneously [7]. Many treatment options are available to strengthen the extensor mechanism system, such as reinforcement to the tibial tuberosity with screws, reinforcement or complete reconstruction of the patella tendon using synthetic ligament, graft substitutes or tendon allograft. Many flaps have been described, particularly lateral gastrocnemius muscle which is a useful local flap reported in many studies and used in our patient [8, 9]. It provides mechanical and biological reinforcement to the extensor apparatus after soft tissue healing is complete [10].

## Conclusion

We have described our staged management of a complex limb injury. These injuries have a huge socio-economic impact. Timely decision making is fundamental in such situations.
